# Cardiovascular coupling during graded postural challenge: comparison between linear tools and joint symbolic analysis

**DOI:** 10.1590/bjpt-rbf.2014.0179

**Published:** 2016-06-30

**Authors:** Alberto Porta, Anielle C. M. Takahashi, Aparecida M. Catai

**Affiliations:** 1 Department of Biomedical Sciences for Health, University of Milan, Milan, Italy; 2 Department of Cardiothoracic, Vascular Anesthesia and Intensive Care, IRCCS Policlinico San Donato, San Donato, Milan, Italy; 3 Departmento de Fisioterapia, Universidade Federal de São Carlos (UFSCar), São Carlos, SP, Brazil

**Keywords:** nonlinear dynamics, head-up tilt, heart rate variability, arterial pressure variability, cardiovascular control, autonomic nervous system

## Abstract

**Background:**

A joint symbolic analysis (JSA) is applied to assess the strength of the cardiovascular coupling from spontaneous beat-to-beat variability of the heart period (HP) and the systolic arterial pressure (SAP) during an experimental protocol inducing a gradual baroreflex unloading evoked by postural change (i.e. graded head-up tilt).

**Method:**

The adopted JSA can quantify the degree of association between the HP and SAP variabilities as a function of the time scale of the HP and SAP patterns. Traditional linear tools assessing the HP-SAP coupling strength, such as squared correlation coefficient, squared coherence function, and percentage of baroreflex sequences, were computed as well for comparison.

**Results:**

We found that: i) JSA indicated that the strength of the cardiovascular coupling at slow temporal scales gradually increased with the magnitude of the orthostatic challenge, while that at fast temporal scales gradually decreased; ii) the squared correlation coefficient and percentage of baroreflex sequences did not detect this behavior; iii) even though squared coherence function could measure the magnitude of the HP-SAP coupling as a function of the time scale, it was less powerful than JSA owing to the larger dispersion of the frequency domain indexes.

**Conclusion:**

Due to its peculiar features and high statistical power, JSA deserves applications to pathological groups in which the link between HP and SAP variabilities is lost or decreased due to the overall depression or impairment of the cardiovascular control.

## BULLET POINTS

- Joint symbolic analysis (JSA) was applied to assess the strength of the cardiovascular coupling during graded orthostatic challenge from spontaneous heart period and arterial pressure variabilities.
- JSA indicated that the strength of the cardiovascular coupling at slow temporal scales gradually increased with the magnitude of the orthostatic challenge, while that at fast temporal scales gradually decreased.
- Since JSA was found superior compared to its linear counterparts, it deserves to be tested in pathological populations likely to exhibit relevant nonlinear interactions between heart period and arterial pressure variabilities.

## Introduction

Symbolic analysis (SA) is a prominent approach utilized in many fields of science to tackle the complexity of the behavior of a dynamical system and describe the interactions among its subparts^1^. SA is based on a coarse graining procedure symbolizing the time course of one (or more) of the variables describing the behavior of the dynamical system. The symbolization procedure creates an alphabet of states that are visited during the functioning of the system. Specific temporal sequences of the various states, associated to particular conditions of the system, are searched and their rate of presence (or absence) is taken as an indication of the likelihood of the system being in the considered condition, thus classifying and labeling specific system behaviors.

In the field of cardiovascular control analysis, univariate SA has been largely applied to heart period (HP) variability given the ability of this approach to identify different states of the autonomic nervous system and quantify its impairment due to pathology^2^ ^-^ ^12^. The univariate SA has been extended to become joint SA (JSA) with the aim of assessing the interactions between two series such as HP and systolic arterial pressure (SAP) variabilities^13^ ^-^ ^17^, HP variability and respiration^17^ ^-^ ^19^, and HP and ventricular repolarization duration variabilities^20^ ^,^ ^21^. Higher levels of integration of neural reflexes can be studied when JSA is gated according to a periodical input such as the respiratory activity^15^.

While the ability of the univariate SA compared to linear methods, such as time and frequency domain analyses, has been demonstrated^4^ ^-^ ^6^ ^,^ ^8^ ^,^ ^10^ ^,^ ^11^, the power of JSA in assessing HP-SAP variability interactions compared to its linear counterparts, such as squared correlation coefficient, squared coherence function, and percentage of baroreflex sequences, is not fully clarified. This capability has been recently suggested by the fact that JSA was found to be helpful in ranking anesthesia procedures according to their ability to preserve cardiovascular control, while the squared coherence function was unable to provide a suitable classification^16^.

The aim of this study was to assess the HP-SAP coupling during graded head-up tilt via JSA together with linear time and frequency domain tools such as squared correlation coefficient, squared coherence function, and percentage of baroreflex sequences. The orthostatic challenge induced modifications of the degree of the HP-SAP association in relation to the magnitude of the challenge (i.e. the tilt table inclination)^22^, thus allowing the comparison of the ability of the methods to track changes in the strength of the cardiovascular coupling.

## Method

### Experimental protocol and data analysis

#### Experimental protocol

A set of recordings collected during an experimental protocol planned to study the effect of a graded postural challenge on the cardiac baroreflex control^22^ were used. Briefly, 19 healthy humans aged from 21 to 48 years (median age=30 years, 8 males) were studied. All subjects had neither history nor clinical evidence of any disease. They had not taken any medication. They refrained from consuming any caffeine or alcohol-containing beverages in the 24 hours before the recordings. Habitual drinkers, smokers and subjects with body mass index larger than 30 kg/m^2^ were excluded from this study. Women under hormone therapy or contraceptive therapy were excluded as well. All experiments were performed in the morning. The subjects lay on the tilt table supported by two belts at the level of thighs and waist respectively and with both feet touching the footrest of the tilt table. During the entire protocol, the subjects breathed spontaneously but they were not allowed to talk. The study was performed according to the Declaration of Helsinki and it was approved by the Human Research Ethics Committee of the “L. Sacco” Hospital and Department of Biomedical and Clinical Sciences, University of Milan, Milan, Italy (approval number 110-09)^22^. A written informed consent was obtained from all subjects.

ECG (lead II) and continuous plethysmographic arterial pressure (Finometer MIDI, Finapres Medical Systems, The Netherlands) were recorded. Signals were sampled at 300 Hz. The arterial pressure was measured from the middle finger of the left hand, being maintained at the level of heart by fixing the subject’s arm to his/her thorax during the upright position. All experimental sessions of the protocol included three periods in the same order: 1) 7 minutes at REST; 2) 10 minutes during passive head-up tilt (T); 3) 8 minutes of recovery. The inclination of the tilt table was randomly chosen within the set {15°,30°,45°,60°,75°,90°} (T15, T30, T45, T60, T75, T90). Each subject underwent one recording with each tilt table inclination and completed the sequence of tilt table angles without experiencing any sign of pre-syncope. The arterial pressure signal was cross-calibrated in each session using a measure provided by a sphygmomanometer at the onset of REST. The auto-calibration procedure of the arterial pressure device was switched off after the first automatic calibration at the onset of the session. Beat-to-beat series extraction was performed after about 2 minutes from the start of each period.

#### Extraction of the beat-to-beat variability series

After detecting all R-waves on the ECG and locating their peak using parabolic interpolation, HP was approximated as the temporal distance between two consecutive parabolic apexes. The maximum of arterial pressure inside of the *i*-th HP [ HP(*i*)], was taken as the *i*-th SAP, SAP(*i*). HP(*i*) and SAP(*i*) were expressed in ms and mmHg respectively. The automatic detections of the R-waves and SAP peaks were visually checked by a trained physician. After extracting the series HP = {HP(*i*), *i*=1,…,*N*} and SAP = {SAP(*i*), *i*=1,…,*N*}, where *i* is the progressive cardiac beat counter and *N* is the total cardiac beat number, *N*=256 consecutive, synchronous, HP and SAP measures were chosen inside the REST and T periods, thus focusing short-term cardiovascular regulatory mechanisms^23^. The stationarity of the selected HP and SAP sequences was tested according to Magagnin et al.^24^ over the original series after linear detrending. If the tests for the steadiness of the mean and variance were not fulfilled, a new random selection was carried out again until the fulfillment of the restricted weak stationarity test^24^.

#### HP-SAP correlation analysis

The squared correlation coefficient of the linear regression in the plane (SAP(*i*),HP(*i*)) was taken as a measure of the degree of the linear coupling between the HP and SAP series in the time domain. This index, indicated as *r* ^2^ in the following, ranges from 0 to 1, where 0 indicates perfect uncorrelation between the HP and SAP, while 1 indicates full association.

#### HP-SAP squared coherence analysis

In the frequency domain, the degree of the linear coupling between the HP and SAP series as a function of the frequency (f) was assessed through the squared coherence function [K^2^(f)], defined as the ratio between the square HP-SAP cross-spectrum modulus divided by the product of the HP and SAP power spectra. K^2^(f) ranges from 0 to 1, where 0 indicates perfect uncorrelation between HP and SAP, while 1 indicates full association. In the calculation of K^2^(f), the cross-spectrum and power spectra were estimated according to the bivariate autoregressive model the coefficients of which were identified via a traditional least squares technique^25^. The model order was fixed at 10. K^2^(f) was sampled in correspondence of the weighted average of the central frequencies of the components found in the SAP series in the low frequency (LF, from 0.04 to 0.15 Hz, 0.15 Hz was included) and high frequency (HF, from 0.15 to 0.4 Hz, 0.15 Hz was excluded) bands, where the weights were the powers of the components estimated from the auto-spectra. The two indexes were referred to as K^2^(LF) and K^2^(HF) in the following.

#### Detection of HP-SAP baroreflex sequences

In the time domain, the strength of the linear HP-SAP coupling can be assessed by evaluating the percentage of baroreflex sequences^26^. A baroreflex sequence is defined as a sequence characterized by the contemporaneous increase or decrease of HP and SAP. The length of the sequences was four cardiac beats (i.e. three HPs). The lag between HP and SAP values, was set to 0 to pick up the fast vagal arm of the baroreflex^27^. When plotted in the plane (SAP(*i*),HP(*i*)), HP and SAP values belonging to a baroreflex sequence lay on a straight line, thus allowing a linear regression analysis. The slope of the regression line was calculated and averaged over all baroreflex sequences. This average was utilized as an estimate of the baroreflex sensitivity (BRS) and expressed in ms/mmHg^26^. A baroreflex sequence was considered to be meaningful if the total HP variation was larger than 5 ms, the total SAP variation was larger than 1 mmHg, and the correlation coefficient in the plane (SAP(*i*),HP(*i*)) was larger than 0.85. The percentage of baroreflex sequences [BRS%], was computed as the ratio of the number of baroreflex sequences to the total number of patterns of the same length multiplied by 100. BRS% ranged from 0 to 1, where 0 indicated perfect HP-SAP uncoupling along the baroreflex pathway, while 1 indicated full association.

#### HP-SAP JSA

**Figure 1:**
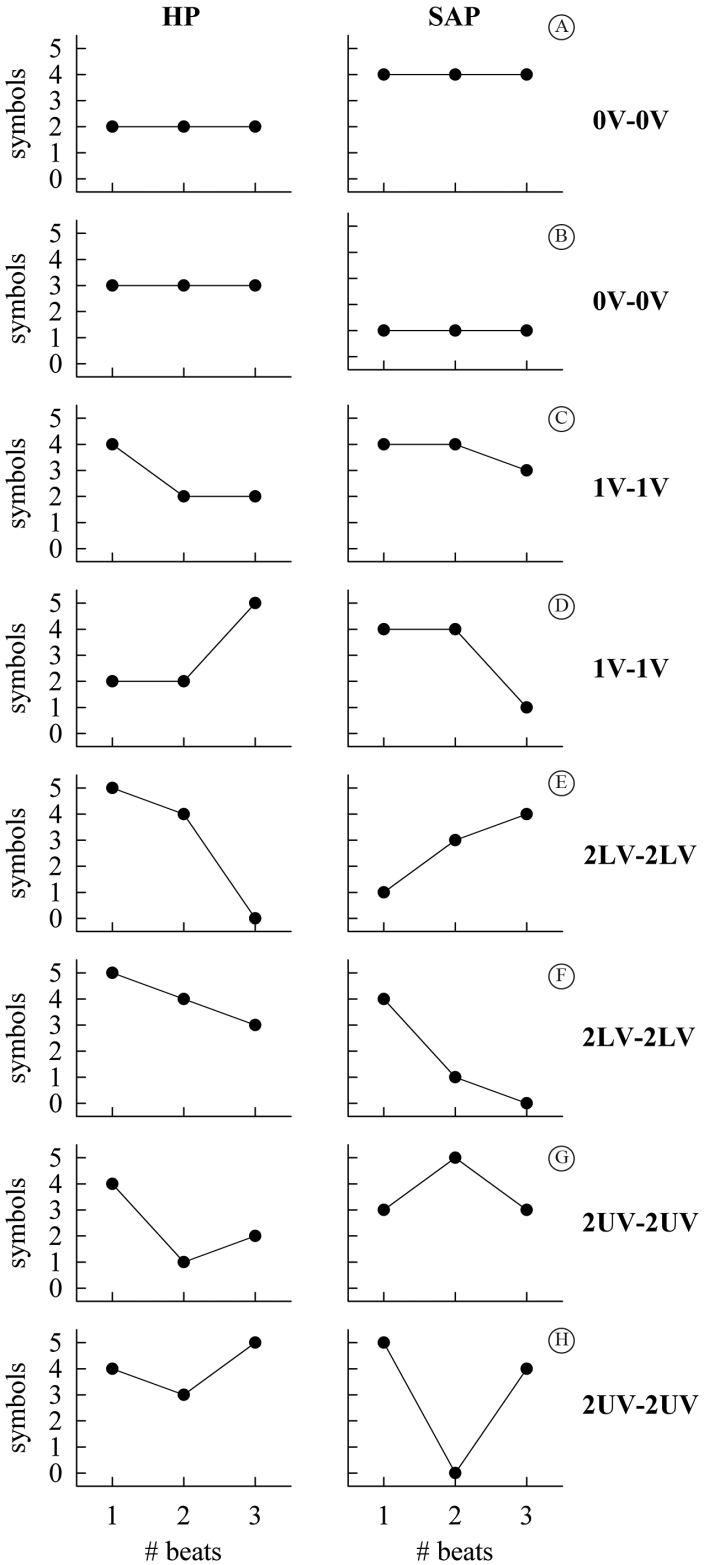
Representation of HP-SAP patterns formed by joining a pattern of HP (left column) with a pattern of SAP (right column). Two illustrative joint patterns for each family are shown: 0V-0V (A, B); 1V-1V (C, D); 2LV-2LV (E, F) and 2UV-2UV (G, H). HP: heart period; SAP: systolic arterial pressure; 0V: no variation; 1V: one variation; 2LV: two like variations; 2UV: two unlike variations.

The univariate SA described in Porta et al.^10^ was applied to symbolize the HP and SAP series and reduce the redundancy of patterns and the JSA set in Porta et al.^15^ was carried out to assess the degree of the HP-SAP coupling. Briefly, the HP and SAP series were transformed into a sequence of ξ=6 symbols. Two sets of symbolic patterns were built by taking three consecutive symbols from the two symbolic series of HP and SAP respectively. All symbolic patterns were classified into four classes depending on the frequency content of the symbolic pattern: i) no variation (0V); ii) one variation (1V); iii) two like variations (2LV); iv) two unlike variations (2UV). 0V and 2UV families collected the most steady and the most variable patterns respectively, while 1V and 2LV classes exhibited an intermediate level of fast frequencies with the 2LV patterns faster than the 1V ones. From the two sequences of HP and SAP symbolic patterns, the JSA created a set of the joint schemes by associating a HP symbolic pattern with the τ-beat-ahead symbolic pattern of SAP, where τ represented the delay between HP and SAP samples. Since the authors were not interested in the causal relation from HP to SAP and vice versa, the delay τ was fixed to 1 according to minimal latency of the HP-SAP closed loop interactions. Indeed, owing to the convention of SAP measurement, SAP(*i*) can affect HP(*i*) along the causal pathway from SAP to HP, while this situation cannot occur along the reverse causal pathway (i.e. from HP to SAP) and one beat delay was imposed^27^. Patterns were defined to be coordinated whether symbolic patterns of HP and SAP belonged to the same family (i.e. 0V, 1V, 2LV and 2UV). All coordinated patterns dropped into four families labeled as 0V-0V, 1V-1V, 2LV-2LV and 2UV-2UV respectively. Two examples of the joint patterns for each pattern family are shown in Figure 1. The percentage of 0V-0V, 1V-1V, 2LV-2LV and 2UV-2UV inside the class of the coordinated patterns was computed and indicated as 0V-0V%, 1V-1V%, 2LV-2LV% and 2UV-2UV%. 0V-0V%, 1V-1V%, 2LV-2LV% and 2UV-2UV% ranging from 0 to 1, where, assigned the type of coordination, 0 indicated perfect HP-SAP uncoupling, while 1 indicated full association.

### Statistical analysis

One way repeated measures analysis of variance (Dunnett’s method for multiple comparisons), or Friedman repeated measures analysis of variance on ranks (Dunnett’s method for multiple comparisons) when appropriate, was applied to check the significance of the differences among parameters compared to REST. The Pearson correlation analysis was performed to check the linear association of any parameter with tilt table inclination. Linear regression analysis was carried out only if a significant difference compared to REST was found. Pearson product moment correlation coefficient *r* and the type I error probability *p* in testing the null hypothesis of flat slope was computed. Statistical analysis was carried out using a commercial statistical program (Sigmaplot, ver.11, Systat Software, San Jose, CA, USA). A *p*<0.05 was always considered as significant.

## Results

**Figure 2:**
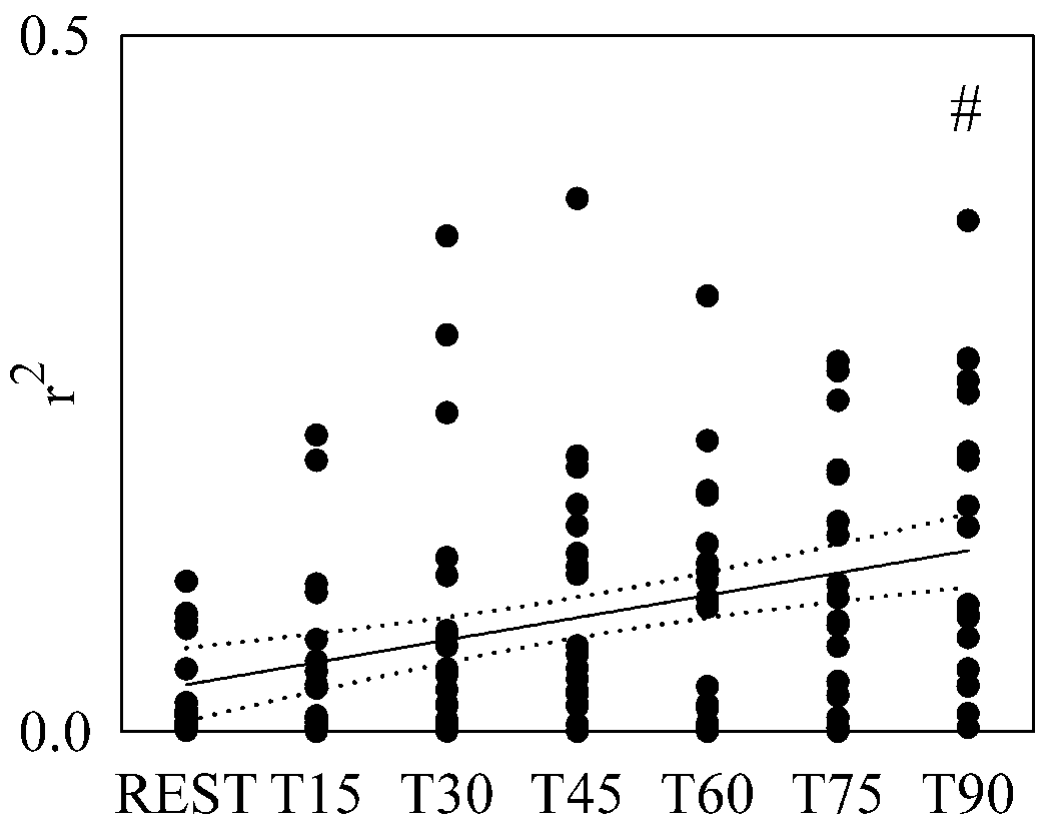
Individual values (solid circles) of r^2^ are shown as a function of the tilt table inclination. The significant difference of the values compared to REST is marked by the symbol #. The linear regression (solid line) and its 95 percent confidence interval (dotted lines) were plotted as well. A significant positive correlation on tilt table angles was found. r^2^: squared correlation coefficient; T: head-up tilt.

Figure 2 shows the individual values (solid circles) of *r* ^2^ as a function of the tilt table inclination. A significant variation of *r* ^2^ with respect to REST was detected only during T90. A significant positive relation of *r* ^2^ on tilt table angles was found (*r*=0.356, *p*=2.62^.^10^-5^). Since a significant modification of *r* ^2^ compared to REST and a significant linear relation of *r* ^2^ on tilt table angles were found, the linear regression (solid line) and its 95 percent confidence interval (dotted lines) were plotted in Figure 2 as well.

**Figure 3:**
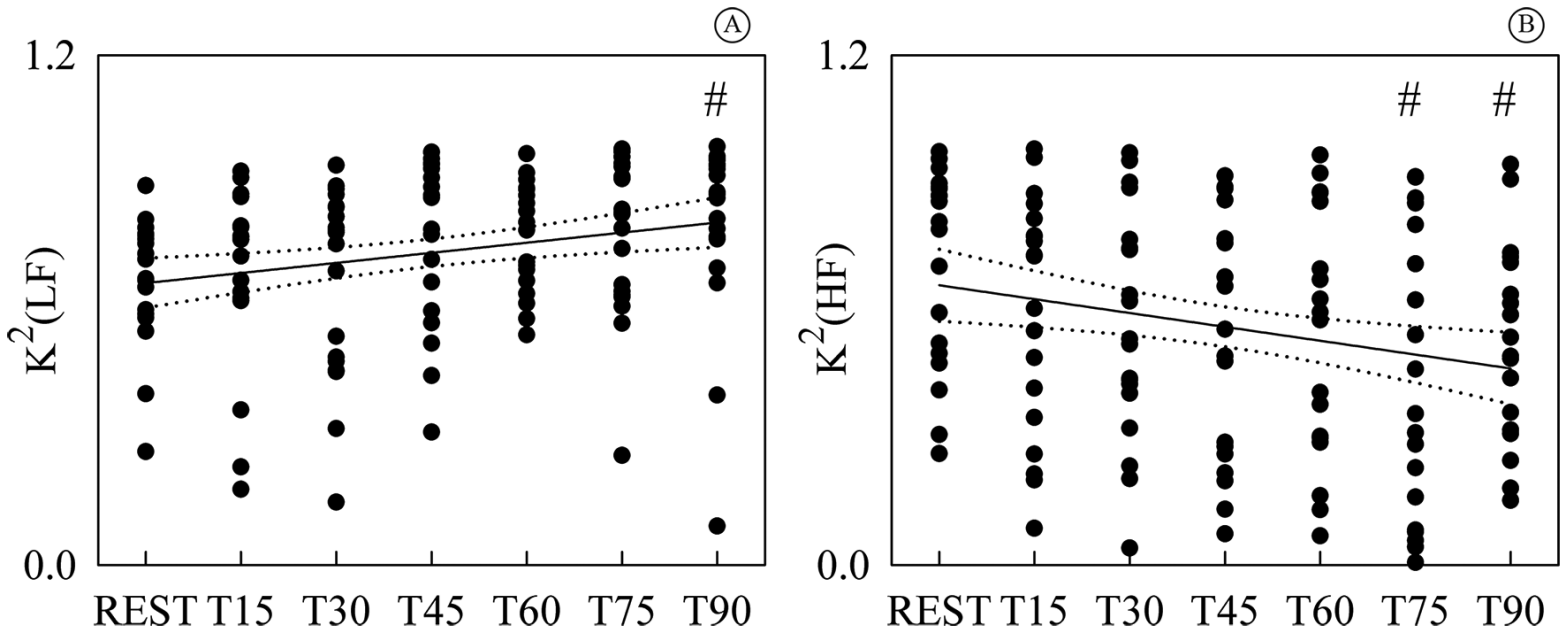
Individual values (solid circles) of K2(LF) (A) and K2(HF) (B) are shown as a function of the tilt table inclination. The significant difference of the values compared to REST is marked by the symbol #. The linear regression (solid line) and its 95 percent confidence interval (dotted lines) were plotted as well. While a significant positive correlation on tilt table angles was found in (A), a negative one was detected in (B). K^2^ (LF): squared coherence at low frequency; K^2^(HF): squared coherence at high frequency; T: head-up tilt.

Figure 3 shows the individual values (solid circles) of K^2^(LF) (Figure 3A) and K^2^(HF) (Figure 3B) as a function of the tilt table inclination. K^2^(LF) was significantly different from REST during T90, while K^2^(HF) was significantly different during T75 and T90. While K^2^(LF) exhibited a positive linear trend with tilt table angles (Figure 3A, *r*=0.245, *p*=4.46^.^10^-3^), K^2^(HF) decreased with the magnitude of the orthostatic challenge (Figure 3B, *r*=-0.234, *p*= 6.81^.^10^-3^). Since a significant modification of K^2^(LF) and K^2^(HF) compared to REST and significant linear relations of K^2^(LF) and K^2^(HF) on tilt table angles were found, linear regressions (solid lines) and their 95 percent confidence intervals (dotted lines) were plotted in Figures 3A, B as well.

**Figure 4:**
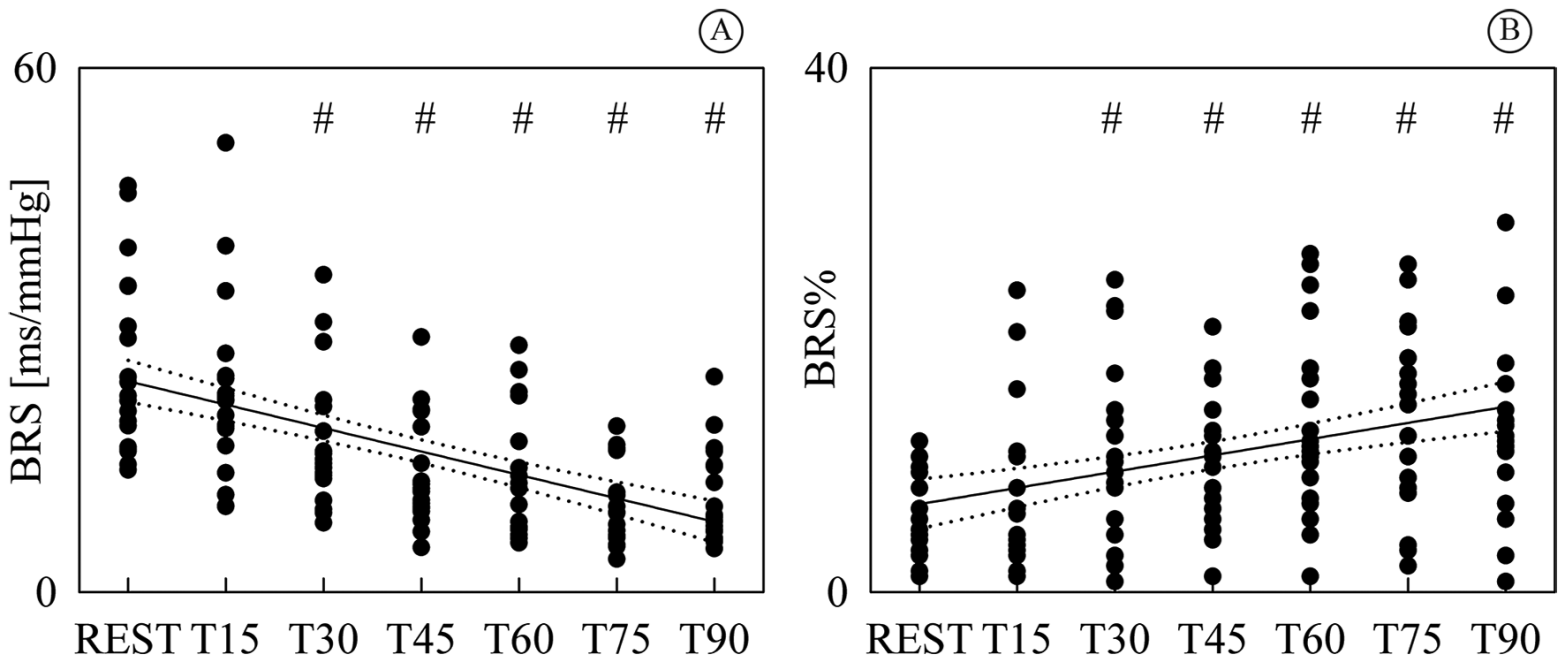
Individual values (solid circles) of BRS (A) and BRS% (B) are shown as a function of the tilt table inclination. The significant difference of the values compared to REST is marked by the symbol #. The linear regression (solid line) and its 95 percent confidence interval (dotted lines) were plotted as well. While a significant negative correlation on tilt table angles was found in (A), a positive one was detected in (B). BRS: baroreflex sensitivity; BRS%: percentage of baroreflex sequences; T: head-up tilt.

Figure 4 shows the individual values (solid circles) of BRS (Figure 4A) and BRS% (Figure 4B) as a function of the tilt table inclination. Both BRS and BRS% were significantly different from REST as early as T30 and steadily varied with tilt table inclination. While BRS exhibited a negative linear trend with tilt table angles (Figure 4A, *r*=-0.575, *p*=4.77^.^10^-13^), BRS% increased with the magnitude of the orthostatic challenge (Figure 4B, *r*=0.378, *p*=7.71^.^10^-6^). Since a significant modification of BRS and BRS% compared to REST and significant linear relations of BRS and BRS% on tilt table angles were found, linear regressions (solid lines) and their 95 percent confidence intervals (dotted lines) were plotted in Figures 4A, B as well.

**Figure 5:**
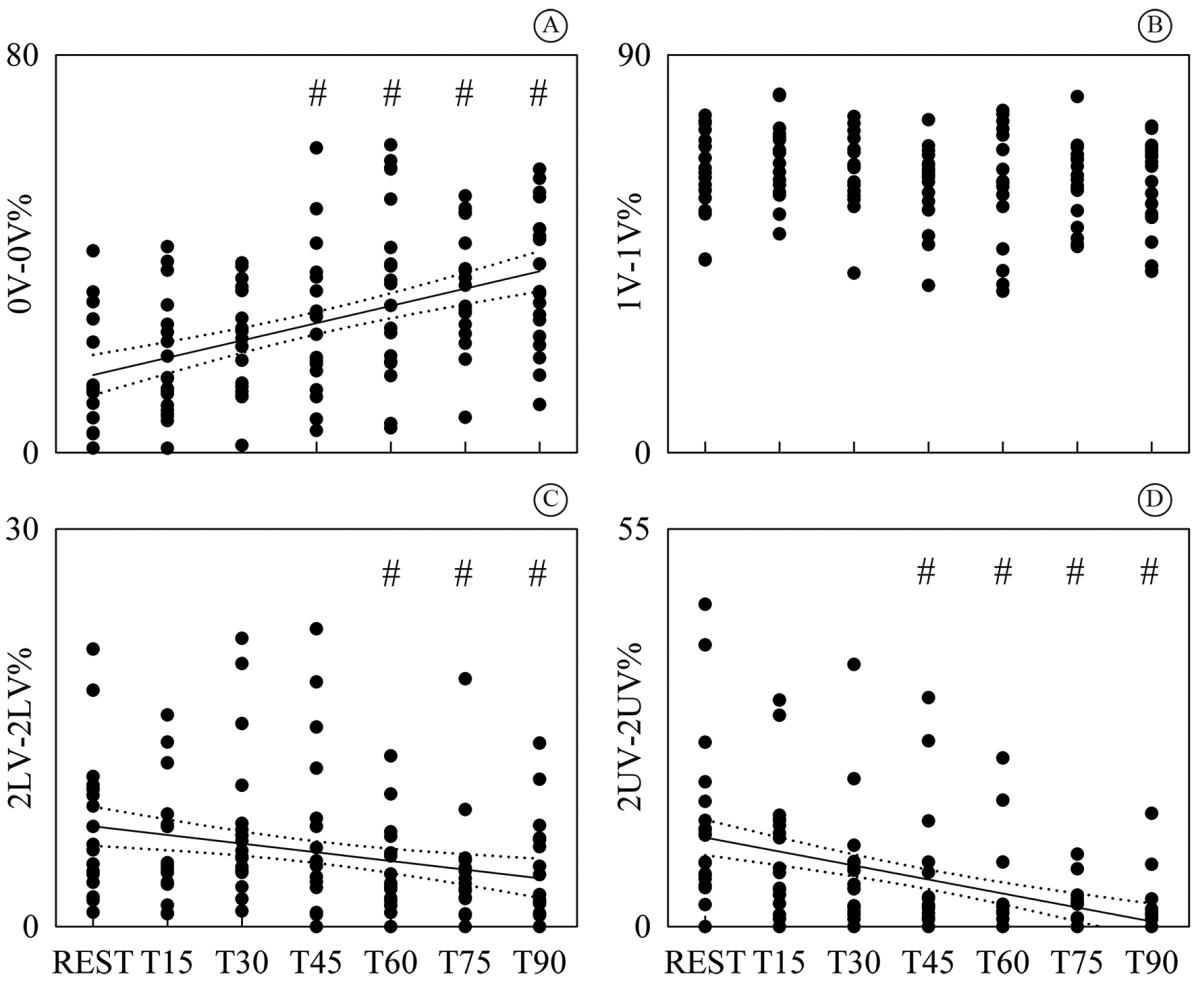
Individual values (solid circles) of 0V-0V% (A); 1V-1V% (B); 2LV-2LV% (C) and 2UV-2UV% (D) are shown as a function of the tilt table inclination. The significant difference of the values compared to REST is marked by the symbol #. The linear regression (solid line) and its 95 percent confidence interval (dotted lines) were plotted as well when a significant variation compared to REST was detected. While a significant positive correlation on tilt table angles was found in (A), a negative one was detected in (C) and (D). No significant difference compared to REST was identified in (B). 0V: no variation; 1V: one variation; 2LV: two like variations; 2UV: two unlike variations; T: head-up tilt.

Figure 5 shows the individual values (solid circles) of 0V-0V% (Figure 5A), 1V-1V% (Figure 5B), 2LV-2LV% (Figure 5C) and 2UV-2UV% (Figure 5D) as a function of the tilt table inclination. 0V-0V% and 2UV-2UV% were significantly different from REST during T45, T60, T75 and T90, while 2LV-2LV% was significantly different during T60, T75 and T90. 1V-1V% did not vary compared to REST. While 0V-0V% increased with tilt table inclination (Figure 5A, *r*=0.471, *p*=1.04^.^10^-8^), 2LV-2LV% and 2UV-2UV% exhibited a negative linear relation on the magnitude of the orthostatic challenge (Figure 5C, *r*=-0.266, *p*=2.0^.^10^-3^ and Figure 5D, *r*=-0.441, *p*=1.08^.^10^-7^). Since 1V-1V% was stable with tilt table angles, the linear regression analysis was not carried out (Figure 5B). Since a significant modification compared to REST and significant linear relations on tilt table angles were found only in the case of 0V-0V%, 2LV-2LV% and 2UV-2UV%, the linear regressions (solid lines) and their 95 percent confidence intervals (dotted lines) were plotted only in Figures 5A, C, D.

It is remarkable that the type I error probability of the linear correlation analysis was much smaller in case of 0V-0V% and 2UV-2UV% than *r* ^2^, K^2^(LF), K^2^(HF) and BRS%, thus suggesting a greater statistical power of JSA than time and frequency domain linear HP-SAP coupling indexes.

## Discussion

The main findings of the study can be summarized as follows: i) the JSA indicated that the strength of the cardiovascular coupling at slow time scales gradually increased with the magnitude of the orthostatic challenge, while coupling at fast time scales gradually decreased; ii) this result could not be achieved by a global time domain index of HP-SAP coupling strength such as the squared correlation coefficient and percentage of baroreflex sequences; iii) the JSA seemed to be more powerful than squared coherence function as a likely result of a more limited dispersion of symbolic indexes.

### JSA of HP and SAP variability series during graded head-up tilt

The adopted JSA could assess the degree of coordination between HP and SAP series at different time scales^15^. This property was the result of the definition of the 0V-0V, 1V-1V, 2LV-2LV and 2UV-2UV families that associate HP and SAP patterns with different frequency contents. For example, the joint pattern 2UV-2UV connected together rapidly changing HP and SAP patterns featuring fast temporal scales, while the joint pattern 0V-0V linked together very stable HP and SAP patterns characterized by slow time scales. The 1V-1V and 2LV-2LV classes allowed the exploration of the degree of HP-SAP coordination at time scales intermediate between those covered by 0V-0V and 2UV-2UV families because 1V and 2LV patterns exhibited a frequency content faster than the 0V class and slower than the 2UV one with the 2LV patterns more rapidly varying than the 1V ones. This study originally showed that the strength of the cardiovascular coupling at slow time scales, as assessed by 0V-0V%, gradually increased with the magnitude of the orthostatic challenge, while that at fast time scales, as assessed by 2UV-2UV%, progressively decreased. The increase of 0V-0V%, with tilt table inclination was likely the effect of an augmented solicitation of the cardiac baroreflex in response to the modification of posture^22^ ^,^ ^28^ ^,^ ^29^, while the decrease of 2UV-2UV% with tilt table angles could have been a consequence of the vagal withdrawal proportional to magnitude of the orthostatic challenge^8^ ^,^ ^30^ ^-^ ^32^ leading to a HP-SAP uncoupling at the respiratory rate.

### JSA is more powerful than its linear counterparts in the assessment of cardiovascular coupling during graded head-up tilt

The considered JSA was more powerful than a traditional linear analysis based on squared correlation coefficient or percentage of baroreflex sequences due to the ability of the symbolic approach to assess the coupling strength at different temporal scales. Indeed, the squared correlation coefficient was a global marker of the HP-SAP association, and the percentage of baroreflex sequences assessed the strength of the variability interactions along the time scale selected for the description of the baroreflex action (here the fast vagal arm). In this specific application, the type I error probability of the squared correlation coefficient was above that of 0V-0V% and 2UV-2UV% probably because the squared correlation coefficient accounted for the opposite trends of the HP-SAP association occurring at faster and slower time scales. Similar conclusion holds for the percentage of baroreflex sequences as a likely consequence of the limited ability of the cardiac baroreflex in accounting for the total HP-SAP interactions^33^ (this index mainly focused the baroreflex ones). It is worth noting that, although the squared correlation coefficient and percentage of baroreflex sequences were indexes spanning different time scales and focused on different mechanisms, the conclusion was exactly the same (i.e. the strength of the HP-SAP coupling increased with the tilt table inclination).

It is remarkable that the considered JSA was more powerful than a frequency domain tool that had intrinsically the ability to measure the coupling strength as a function of the time scale such as the squared coherence function. Indeed, in this experimental protocol squared coherence function in the LF and HF bands exhibited the same trends with the magnitude of the orthostatic stimulus as 0V-0V% and 2UV-2UV% indexes respectively. However, the type I error probability of the squared coherence function in the LF and HF bands was much higher than that of 0V-0V% and 2UV-2UV%. This worse performance was the consequence of a larger within-subject and between-subject dispersion of the frequency domain indexes. The smaller dispersion of JSA markers compared to frequency domain indexes might have been the effect of the coarse graining procedure reducing the superposed noise^1^. However, the capability of the JSA to interpret nonlinear dynamics may also have played a role in boosting performances.

### Perspective and significance

The degree of the cardiovascular coupling was usually significant in healthy subjects due to the closed-loop interactions between HP and SAP variability. Both mechanical feedforward, due to diastolic runoff and the Starling law, and baroreflex feedback contributed to the intertwined relations between HP and SAP variabilities^27^ ^,^ ^33^. Therefore, a lack of association between HP and SAP variability or even a decrease of cardiovascular coupling below the high values usually found at REST in healthy subjects was considered to be an index of impairment of the cardiovascular control. For example, the strength of the cardiovascular coupling decreased, especially at slow time scales, when cardiovascular control was depressed by general anesthesia^34^. Pathological conditions such as heart failure, myocardial infarction, cardiac autonomic neuropathy, peripheral neuropathy, fibromyalgia, dysautonomia, orthostatic intolerance and frailty syndrome significantly decreased the magnitude of the HP-SAP coupling to such a level that HP and SAP series might be uncoupled^35^ ^-^ ^39^. However, even an exaggerated increase in coordination between HP and SAP variabilities could indicate the impairment of the cardiovascular control. For example, just before the development of syncope, the degree of HP-SAP association at slow time scales was found to be increased^15^. In the context of the quantification of the strength of the HP-SAP variability interactions, JSA seems to be a powerful method capable of coping with the usually large within-subject and between-subject variability present in pathological groups and in particularly challenging situations such as during general anesthesia and in critical care units. In addition, the application of this tool might provide additional information compared to linear methods when nonlinear HP-SAP variability interactions are present^40^.
